# Microscale Sensor Arrays for the Detection of Dopamine Using PEDOT:PSS Organic Electrochemical Transistors

**DOI:** 10.3390/s24165244

**Published:** 2024-08-14

**Authors:** Chunling Li, Yingying He, Sven Ingebrandt, Xuan Thang Vu

**Affiliations:** Institute of Materials in Electrical Engineering 1, RWTH Aachen University, Sommerfeldstr. 24, 52074 Aachen, Germany; chunling.li@iwe1.rwth-aachen.de (C.L.); yingying.he@rwth-aachen.de (Y.H.); ingebrandt@iwe1.rwth-aachen.de (S.I.)

**Keywords:** PEDOT:PSS, organic electrochemical transistor, dopamine, real-time measurement, chitosan

## Abstract

We present a sensor array of microscale organic electrochemical transistors (OECTs) using poly (3,4−ethylenedioxythiophene):poly(styrenesulfonic acid) (PEDOT:PSS) as the channel material. The devices show high sensitivity and selectivity to detect dopamine (DA) with platinum (Pt) as a pseudo−reference gate electrode. First, we describe the wafer−scale fabrication process for manufacturing the PEDOT:PSS OECTs, and then we introduce a dilution method to adjust the thickness of the PEDOT:PSS film. Next, we investigate the effect of the film thickness on the sensitivity of DA detection. Reducing the film thickness enhances the sensitivity of DA detection within the concentration range of 1 μM to 100 μM. The OECTs show impressive sensitivitywith a limit of detection (LoD) as low as 1 nM and a high selectivity against uric acid (UA) and ascorbic acid (AA). Finally, we modify the surface of the Pt gate electrode with chitosan to improve the selectivity of OECTs at high concentrations of up to 100 µM to expand the detection range.

## 1. Introduction

Dopamine (DA) is a neurotransmitter found in the central nervous system (CNS), renal system, cardiovascular system, and other areas [1]. DA affects various neurological and peripheral activities, including motor control, cognition, reward, blood pressure, hormone release, and sodium balance [2]. DA is produced by dopaminergic neurons in the substantia nigra region of the brain and then transferred to the whole body. For a healthy person, the concentration of dopamine is 0–0.25 nM in blood and 0.3–3 µM in urine [3]. Abnormal levels of DA in the body can cause various physiological disorders. High levels of DA can indicate cardiotoxicity, which can lead to rapid heart rates and heart failure [4]. Conversely, low levels of DA in the central nervous system can result in several diseases, such as Parkinson’s disease, Alzheimer’s disease, and depression [5]. In recent years, sensors have been developed in various formats for highly sensitive and selective detection of DA in vivo and in vitro. Such sensor assays play a crucial role in clinical diagnosis, disease prevention, and monitoring treatment efficacy [6]. However, the detection of DA is challenging due to its low concentration in body fluids and the interference of ascorbic acid (AA) and uric acid (UA) in many assays, which have electrochemical properties similar to DA.

Numerous detection methods were developed for DA including high-performance liquid chromatography (HPLC) [7], capillary electrophoresis [8], mass spectroscopy [9], ultraviolet–visible spectrophotometry [10], fluorescence spectrometry [11], and electrochemical methods [12,13,14]. HPLC and mass spectroscopy offer remarkably low detection limits. However, they require expensive specialized instruments, pose a long response time, and need complicated operational procedures. Electrochemical methods, such as amperometry [15], cyclic voltammetry (CV) [16], and differential pulse voltammetry [17], have emerged as promising alternatives. The quantification of the DA level is achieved through measuring the currents generated by the oxidation of DA. Although these approaches exhibit rapid response, simplicity, and portability, they face challenges in selectively detecting DA in the presence of AA and UA, which can undergo oxidation at close potentials [18].

Over recent years, organic electrochemical transistors (OECTs) have been successfully used in various biosensing applications owing to their remarkable signal amplification, straightforward architecture, and flexibility [19,20,21]. An OECT typically consists of three electrodes: source, drain, and gate. The polymeric transistor channel is contacted by metallic source and drain contacts, while the gate electrode is utilized to establish an electrical contact with the electrolyte solution [22,23,24]. PEDOT:PSS has gained significant research attention [19,25,26] thanks to its outstanding properties, such as high electrical conductivity, transparency, flexibility, and biocompatibility [27,28,29]. PEDOT:PSS is a p−type organic semiconductor and is commonly integrated as channel material into OECTs [30]. This configuration makes PEDOT:PSS OECTs highly promising for a wide range of biosensing applications, including H_2_O_2_ monitoring [31], glucose detection [22,32], and cell recording [26].

Several technologies are available to fabricate OECTs, including screen printing, spray printing, inkjet printing, and microfabrication processes [26,33,34,35]. Although printing technologies offer speed, simplicity, cost−effectiveness, and efficiency, their suitability for reproducible fabrication of OECT arrays is limited by low spatial resolution and unstable material quality [36,37,38]. As a result, these techniques are primarily recommended for large−scale OECT applications with lower resolution requirements. According to the OECT operation model developed by Bernards and Malliaras [39], the transconductance (g) represents the signal amplification of the OECTs and has a linear relationship with the channel geometry (Wd/L), where W, d, and L represent the channel width, thickness, and length, respectively. In addition, the channel geometry and, hence, the volume of the PEDOT:PSS material significantly affects the channel capacitance and transient response time of the transistors [20,40]. Therefore, the channel geometry and the PEDOT:PSS thickness of OECTs play a critical role in the electrical characteristics. Miniaturization of the channel geometry leads to improved electrical characteristics in certain scenarios. Hence, microfabrication technologies, which provide precise control over the channel area, are critical for the fabrication of high−performance OECTs. These microscale OECTs have been used in diverse applications, including wearable electronics, neural interface devices, and biosensors.

One of the challenges of DA detection is the interference from molecules like AA and UA, which are commonly present in biological samples and typically more abundant. These molecules can potentially impact DA detection, leading to inaccurate readings. To address this challenge, many modification methods need to be performed to improve selective detection, ensuring that the detected signal specifically represents DA concentrations. Based on the device structure, the OECTs include two individual components: the PEDOT:PSS channel part and the gate electrode part. Many problems arise with the direct chemical modification of the PEDOT:PSS channel, such as disrupted conductivity and limited and non-uniform distribution of bioreceptors [41]. Therefore, the modification of the gate electrode in contact with the electrolyte and separated from the PEDOT:PSS channel has gained more and more interest. The chemical modification of the gate electrode does not affect the performance of polymeric channel material [42,43]. Pt is often utilized as the gate electrode for DA detection due to its strong electro−oxidation ability. However, the selectivity of Pt is relatively weak, especially in the case of high concentrations of interfering molecules. Therefore, it is necessary to modify the Pt electrode for selective detection of the desired analyte. Several research groups have recently employed PEDOT:PSS OECTs for DA detection. Tang et al. demonstrated highly sensitive DA biosensors based on PEDOT:PSS OECTs with different gate electrodes (graphite, Au, and Pt electrodes). Among these gate electrodes, the Pt gate electrode exhibited an LoD for DA at approximately 5 nM [44]. Liao et al. reported that modifying the surface of the Pt gate electrode can significantly enhance the selectivity of OECT−based dopamine sensors. In their study, OECTs with Nafion (1.0%)–graphene/Pt gate electrodes showed sensitivity to DA detection from 5 nM to 1 µM in the presence of interfering AA and UA [45]. Table 1 illustrates the current state of the art of PEDOT:PSS−based OECTs for DA detection. 

In this work, we report highly sensitive and selective DA detection by employing micro−fabricated PEDOT:PSS OECTs with a Pt gate electrode. First, the microfabrication process for the PEDOT:PSS OECT microarrays is presented. This included several steps of optical lithography, lift-off, and etching. Ultra−thin PEDOT:PSS films with various thicknesses were used as the channel material of the OECTs. Subsequently, a systematic analysis was carried out to determine how variations in PEDOT:PSS thickness impact the sensitivity of the OECTs for DA detection. In addition, real-time measurements for DA detection with the OECT microarrays were performed with concentrations ranging from 1 nM to 3 mM. Following this, the highly selective detection of DA against interfering concentrations of AA and UA was conducted with a concentration range of 1 nM to 100 μM. Finally, the surface of the Pt gate electrode was modified by the chitosan to improve the selective detection of DA. 

## 2. Materials and Methods

### 2.1. Working Principle of Dopamine Detection Using PEDOT:PSS OECTs

**Figure 1:**
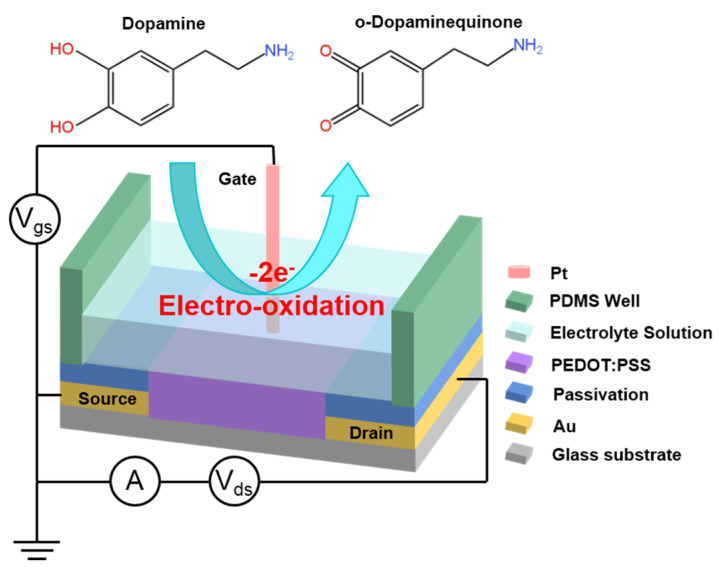
Schematic of the working principle for DA detection using PEDOT:PSS OECTs [44,45]. The arrow represents the electro−oxidation reaction of DA at the surface of a gate electrode.

The working principle of DA detection using PEDOT:PSS OECTs has been described previously [44,45]. Figure 1 presents the schematics of the working principle for DA detection using PEDOT:PSS OECTs with a Pt electrode. The DA in the electrolyte solution undergoes electro−oxidization to o−dopaminequinone at the surface of the Pt electrode. The Faradic current generated by the electro-oxidation of DA reduces the potential drop at the electrolyte/gate interface, thereby raising the effective gate voltage for the transistor. The effective gate voltage is given by the following equation [44,45]:

(1) $$V_{g}^{eff}=V_{G}+2.30\left(1+\gamma \right)\frac{kT}{2q}\log\left[C_{DA}\right]+A$$

where *γ = C_C_/C_G_* is the ratio between the capacitances of the channel/electrolyte interface (*C_C_*) and the gate/electrolyte interface (*C_G_*); *k* is the Boltzmann constant; *T* is the Kelvin temperature; [*C_DA_*] is the molar concentration of DA; and *A* is a constant.

As a result, the concentration of DA affects the effective gate voltage, leading to a variation in the drain current of the OECTs. The drain current is given by [45]

(2) $$I_{ds}=\frac{qµp_{0}dW}{LV_{p}}\left(V_{p}-V_{g}^{eff}+\frac{V_{ds}}{2}\right),\;\left(\left|V_{ds}\right|\ll \left|V_{p}-V_{g}^{eff}\right|\right)\;V_{p}=\frac{qp_{0}t}{c_{i}},\;V_{g}^{eff}=V_{G}+V_{offset}$$

where q is the electron charge; µ is the hole mobility; $p_{0}\;$ is the initial hole density; d is the thickness of the conductive polymer layer; W and L are the width and the length of the channel area of the OECT device, respectively; $V_{p}$ and $V_{g}^{eff}$ are the pinch-off voltage and the effective gate voltage, respectively; $c_{i}\;$ is the effective gate capacitance per unit area; and $V_{offset}$ is the offset voltage on the surface of the gate or the channel.

Therefore, the modulation of the channel current by DA is primarily caused by the change in the effective gate voltage given by Equation (1). As the concentration of DA increases, $V_{g}^{eff}$ also increases, resulting in a reduction in $I_{ds}$ in the channel, as described by Equation (2).

### 2.2. Materials 

PEDOT:PSS solution (Clevios PH 1000) was purchased from Heraeus, Germany. Ethylene glycol (EG), 3−Glycidyloxypropyl-triethoxysilan (GOPS), chitosan, dopamine hydrochloride (DA), ascorbic acid (AA), uric acid (UA), and phosphate-buffered saline (PBS) solution (pH = 7.4) were all purchased from Sigma-Aldrich, Taufkirchen, Germany. Acetic acid (100%) was purchased from Merck KGaA Company. Dopamine hydrochloride (DA) was stored at 4 °C when not in use. The other chemicals were stored at room temperature. 

### 2.3. Preparation of PEDOT:PSS Solution and Analyte Solutions

#### 2.3.1. PEDOT:PSS Solution

The PEDOT:PSS solution was initially filtered using a 200 nm syringe filter (Luer Solo, B. Braun, Melsungen AG, Germany) to remove larger particles. Subsequently, 5 mL of the filtered PEDOT:PSS solution was mixed with 250 μL of EG and 43 μL of GOPS to create PEDOT:PSS mixture solution A [52,53]. Mixture solution A was then diluted with DI water in ratios of 1:2, 1:4, and 1:8 (*v*/*v*) to obtain PEDOT:PSS mixture solutions B, C, and D, respectively.

#### 2.3.2. Analyte Solutions

First, 18.964 mg of DA powder was added to 1000 µL of PBS solution to obtain the 3 mM DA solution. Different concentrations of DA solutions (1 mM, 300 µM, 250 µM, 100 µM, 30 µM, 10 µM, 3 µM, 1 µM, 100 nM, 10 nM, and 1 nM) were then obtained by gradually diluting the 3 mM stock solution with PBS. Similarly, solutions of ascorbic acid (AA) and uric acid (UA) ranging from 1 nM to 100 µM were prepared using the same procedure and stored at 4 °C when not in use. To make a 1.0% chitosan solution, 1.0 g of chitosan powder was added to 100 mL of 1.0% acetic acid and stirred for 24 h at room temperature. The prepared chitosan solution was also stored at 4 °C when not in use.

### 2.4. Fabrication of OECTs

**Figure 2:**
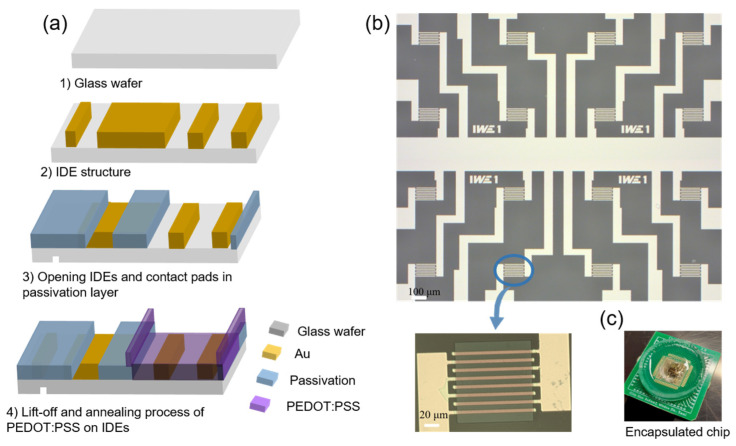
(**a**) Fabrication process: Step 1. Lithography for Au patterning. Step 2. Lift−off for interdigital electrodes (IDEs). Step 3. Dry etching to open IDEs and contact pads. Step 4. Lift−off and annealing process of PEDOT:PSS on IDEs. (**b**) Microscopy image showing 16 OECT channels and a single OECT. (**c**) An encapsulated chip.

The wafer−scale device fabrication process for the OECTs is shown in Figure 2. In the first step, interdigitated electrode arrays (IDEs) were fabricated on 100 mm diameter borosilicate glass wafers (Microchemicals GmbH, Ulm, Germany) by optical lithography and lift−off. IDEs were used for the contacts for PEDOT:PSS to increase the W/L ratio of the transistors. The IDEs were made from 100 nm Au with 5 nm of Cr as an adhesion layer using electron beam evaporation. As shown in Figure 2b, the single chip has a dimension of (7 × 7) mm^2^ and includes 16 individual addressable transistor channels in a common source configuration. A 300 nm thick silicon oxide passivation layer was applied to the entire wafer with a plasma−enhanced chemical vapor deposition (PECVD) process. The contact pads and sensing areas were opened through a reactive−ion etching (RIE) process. The wafer was then half−diced from the back side in order to separate the chips after depositing the PEDOT:PSS. Afterward, the PEDOT:PSS area was structured through a lithography process. Prior to spin−coating the PEDOT:PSS solution, the wafers were cleaned and activated using oxygen plasma (300 W, 300 sccm) for 1 min. The PEDOT:PSS/EG/GOPS mixture solution was spin−coated using a spin−coater system (Sawatec AG, Sax, Switzerland) on the IDE arrays at 4000 rpm and baked at 110 °C for 1 min. Subsequently, the lift−off process was carried out using acetone, and the wafers were then annealed at 140 °C for 1 h. 

The chips could be separated by breaking the glass substrate. Finally, the chips were wire−bonded and encapsulated for electrical characterization and DA detection. Figure 2b presents a complete 16−channel OECT device along with a microscope image of a single channel. An encapsulated chip is shown in Figure 2c.

### 2.5. Cyclic Voltammetry Characterization 

For additional experiments detecting DA in an electrochemical assay using cyclic voltammetry, the working electrode (WE) and the counter electrode (CE) were Pt wires, while the reference electrode (RE) was an Ag/AgCl electrode. These three electrodes were all immersed in an electrolytic cell with the DA solution. The voltage was swept linearly from −0.2 V to +0.8 V with the scan rate of 0.05 V/s between the WE and the RE, then reversed from +0.8 V to −0.2 V. This process was repeated multiple times, and the current was recorded between the WE and the CE. 

### 2.6. Electrical Characterization of Micro OECT Arrays

Electrical characterization was facilitated with a semiconductor characterization system (4200A-SCS, Keithley Instruments, Solon, OH, USA). A two−probe resistance measurement technique was used to characterize the resistance of the fabricated OECTs with different thicknesses of PEDOT:PSS thin films in air. A voltage sweep ranging from −0.1 V to +0.1 V was systematically applied to the source and drain electrodes. The resulting resistance was determined by calculating the slope of the linear IV curve through the formula R = V/I, where V and I represent the applied voltage and the measured current value, respectively. The transfer characteristics of the fabricated OECTs were evaluated in different concentrations of analyte solutions. A Pt electrode was used as the electrolyte gate electrode. A gate−source voltage (V_GS_) sweep ranging from −0.4 V to +0.8 V with a step of 0.04 V was systematically applied. Additionally, the drain–source voltage (V_DS_) with a constant value of −0.3 V was applied. For the real-time sensing of DA concentrations, V_DS_ at −0.3 V and V_GS_ at +0.3 V were chosen as the working points. The drain current value was acquired at an interval of 0.5 s. 

## 3. Results

### 3.1. Thickness of PEDOT:PSS Film

**Figure 3:**
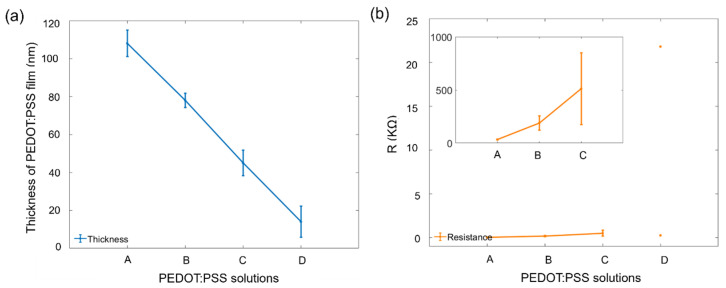
(**a**) Thicknesses of PEDOT:PSS films and (**b**) resistances between source and drain electrodes after depositing PEDOT:PSS mixture solutions A, B, C, and D on the OECTs.

The PEDOT:PSS mixture solutions A, B, C, and D were prepared as described in Section 2.3.1. After depositing PEDOT:PSS on OECTs, the thicknesses of PEDOT:PSS films were determined by measuring the step height with a stylus profilometer (DektakXT, Bruker, Berlin, Germany) on multiple points distributed on the OECT. Figure 3a presents the thickness variations in the PEDOT:PSS films, which were obtained by using the different PEDOT:PSS solutions. The results show that PEDOT:PSS mixture solutions A, B, C, and D resulted in film thicknesses of 108 ± 7 nm, 78 ± 4 nm, 45 ± 7 nm, and 14 ± 8 nm, respectively. The resistance between source and drain electrodes was further measured by applying a voltage sweep ranging from −0.1 V to +0.1 V, and the results are displayed in Figure 3b. The resistance values of 33 ± 2 Ω, 188 ± 66 Ω, and 512 ± 200 Ω were obtained for the PEDOT:PSS mixture solutions A, B, and C, respectively. After depositing PEDOT:PSS mixture solution D on one chip, only two channels could achieve the linear IV curve, and the resistance of these two channels exhibited significant differences. Thus, the electrical characteristics of the OECTs for DA detection were evaluated using PEDOT:PSS film thicknesses of 108 ± 7 nm, 78 ± 4 nm, 45 ± 7 nm, and 14 ± 8 nm. 

### 3.2. CV Characterization of Dopamine Detection

**Figure 4:**
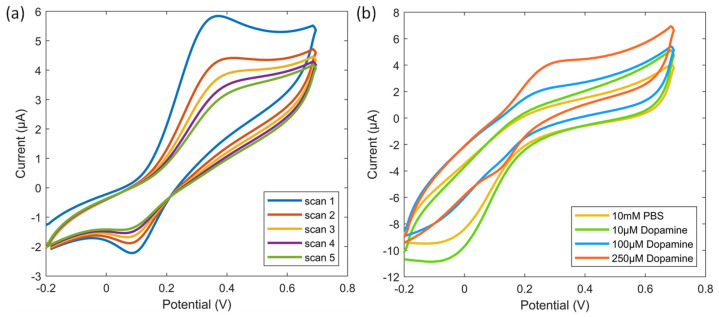
CV characteristics with a voltage sweep from −0.2 to +0.8 V at a scan rate of 0.05 V/s, then reversed (WE and CE: Pt electrodes, RE: Ag/AgCl electrode). (**a**) CV curves from five continuous scans with 1 mM DA solution applied. (**b**) CV curves when adding different DA concentrations.

Figure 4a displays the CV characterization of 1 mM of DA solution with five continuous scans. The voltage was swept from −0.2 to +0.8 V, then reversed. When the voltage swept from −0.2 to +0.8 V, the recorded current first increased rapidly until the peak was reached, where DA underwent electro-oxidation at the Pt working electrode. The oxidation peak currents were observed between +0.2 V and +0.4 V. After the oxidation peak, the rising trend of the current slowed or remained constant, then accelerated again as the scan approached the voltage limit (+0.8 V). When the voltage was swept reversely (from +0.8 to −0.2 V), the reduction peak currents were observed between 0 and +0.2 V, indicating the electro-reduction of DA at the Pt working electrode. As the number of scans increased, the recorded currents decreased until stable currents were obtained after four continuous scans. These results indicate a decrease in DA concentration available for the electro-reoxidation. Figure 4b displays the CV characterization of DA solutions with different concentrations from 10 µM to 250 µM. The characterizations were carried out using the same experimental procedure, respectively. The CV characterization of each concentration was continuously scanned five times, and the fourth scan of each curve was extracted for comparison. The oxidation peak current was observed between 0.2 V and 0.4 V only at DA concentrations of 100 µM and 250 µM. This indicates that the Pt working electrode did not exhibit any significant current response until 100 µM of DA was added.

### 3.3. Electrochemical Characterization of OECT Microarrays for DA Detection

**Figure 5:**
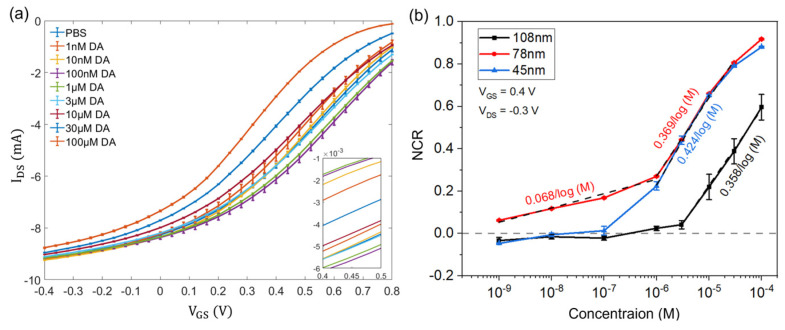
(**a**) Transfer characteristics of PEDOT:PSS OECTs when various DA concentrations are applied (n = 3); V_GS_ is from −0.4 V to 0.8 V and V_DS_ is −0.3 V. (**b**) Normalization plot of the OECT responses versus the DA concentrations with three different PEDOT:PSS thicknesses; V_GS_ = 0.4 V and V_DS_ = −0.3 V.

As illustrated in Figure 5a, the transfer characteristics of the OECTs were evaluated at different concentrations of DA solution using a Pt electrode as the pseudo−reference electrode. V_GS_ was swept from −0.4 V to +0.8 V, and V_DS_ was maintained constantly at −0.3 V. Each transfer curve was measured continuously three times, and every transfer curve shows the depletion mode of transistor performance. Thus, the transistor is normally in its “ON” state at the zero-gate voltage, and the drain current will decrease with increasing gate voltage between the source and gate electrodes. The results indicate that increasing DA concentrations lead to a shift of the transfer characteristics at the oxidation potential of DA (between 0.3 V and 0.4 V) to the direction of more negative voltages. Due to the electro-oxidation of DA at the Pt electrode, a Faradic current is produced, which increases the effectively applied gate voltage and results in a change in the transfer characteristics of the OECTs.

The normalized response of source–drain current (NCR) is extracted from the transfer characteristics and plotted as a function of analyte concentrations. Normalization is calculated relative to the drain current without analyte as follows:

(3) $$\mathrm{NCR}=\frac{I_{DS}^{con=0}-I_{DS}}{I_{DS}^{con=0}}$$

where $I_{DS}^{con=0}$ is the drain current at zero concentration (without analyte), and *I_DS_* is the drain current at a higher concentration of the analyte. The sensitivity of analyte detection is determined by calculating the slope of the NCR curve over the analyte concentrations in a logarithmic scale, allowing for comparison between different OECTs. 

### 3.4. Effect of the PEDOT:PSS Film Thickness for the Sensitivity of DA Detection

Experiments were carried out to study the sensitivity of OECTs with three different thicknesses of PEDOT:PSS (108 nm, 78 nm, and 45 nm) in a PBS solution with dopamine (DA) concentrations ranging from 1 nM to 100 µM. A Pt electrode was used as the pseudo-reference electrode for all characterizations. The gate voltage (V_GS_) was swept from −0.4 V to +0.8 V, while the drain–source voltage (V_DS_) was maintained at −0.3 V throughout. The transfer characteristics of OECTs with each PEDOT:PSS thickness showed depletion mode transistor performance. Furthermore, an increase in DA concentration resulted in a shift of the transfer curve towards more negative voltages.

Figure 5b presents the relationship between NCR and DA concentrations ranging from 1 nM to 100 µM at a V_GS_ of 0.4 V and a V_DS_ of −0.3 V. NCR of OECTs with each PEDOT:PSS thickness is obtained from their transfer characteristics, respectively. The black curve represents the relationship between NCR and DA concentrations using the OECTs with 108 nm of PEDOT:PSS. These OECTs display a linear relation between NCR and DA concentrations ranging from 3 µM to 100 µM and exhibit a sensitivity of 0.358/log (M). The red curve shows the dependence of NCR and DA concentrations with the OECTs based on 78 nm thickness of PEDOT:PSS, while the blue curve displays the relation of NCR and DA concentrations when 45 nm of PEDOT:PSS were used for the OECTs. The NCR changes in the transistors with 78 nm of PEDOT:PSS show a linear relationship with the DA concentrations on a logarithmic scale within the concentration range of 1 µM to 30 µM, and the sensitivity is 0.369/log (M). The NCR changes in the transistors with 45 nm of PEDOT:PSS display a linear relationship with the DA concentrations on a logarithmic scale within the concentration range of 1 µM to 30 µM, while the OECTs show a sensitivity of 0.424/log (M). The results in Figure 5b indicate that the reduction in the PEDOT:PSS thickness will lead to an increase in the sensitivity of the OECTs for DA detection within the concentration range of 1 µM to 30 µM. When the thickness of PEDOT:PSS film reduces, the channel capacitance (*C_channel_*) will decrease. As a result, the ratio of gate capacitance (*C_gate_*) to channel capacitance (*C_channel_*) increases, leading to more efficient gating and better sensitivity of the OECTs [54]. 

As shown in Figure 5b, the transistors with 45 nm and 108 nm of PEDOT:PSS show an LoD of 100 nM and 3 µM, respectively. The OECTs with 78 nm thickness of PEDOT:PSS exhibit the LoD as low as 1 nm. Additionally, these OECTs depositing 78 nm of PEDOT:PSS display a linear function between NCR and DA concentrations ranging from 1 nm to 1 µM with a sensitivity of 0.068/log (M). Therefore, we selected the optimized PEDOT:PSS thickness of 78 nm for the selectivity characterization of the sensors in the following.

### 3.5. Selectivity of DA Detection against AA and UA Using OECT Microarrays

We evaluated the transfer characteristics of the OECTs based on 78 nm of PEDOT:PSS thickness in AA and UA solutions with varied concentrations (from 1 nM to 100 µM) using the Pt electrode, respectively. In these measurements, V_GS_ was swept from −0.4 V to 0.8 V, and V_DS_ was kept constantly at −0.3 V. Each transfer curve was measured continuously three times, and all the transfer curves exhibited a depletion mode transistor performance. The transfer curves of AA and UA show only a small shift in the direction of more negative voltages, especially when lower concentrations were applied. Furthermore, the NCR of AA and UA detection was extracted from their transfer characteristics and plotted as a function of concentrations on a logarithmic scale within the range of 1 nM to 100 µM. 

**Figure 6:**
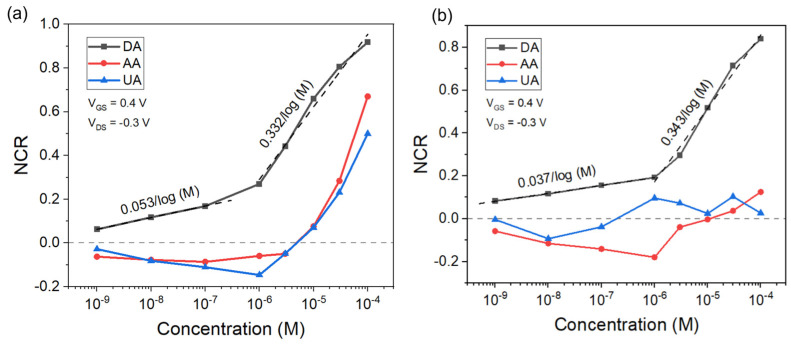
Normalization plots of the OECT responses versus the analyte concentrations show the selectivity of the OECTs for DA against AA and UA before (**a**) and after (**b**) modifying the surface of Pt electrode with chitosan. V_GS_ = 0.4 V and V_DS_ = −0.3 V. The dashed lines represent linear fits line to get the sensitivity in the indicated concentration range.

Figure 6a displays the NCR plot of the OECTs versus the analyte concentrations at a V_GS_ of 0.4 V and a V_DS_ of −0.3 V. The black curve represents the relationship between NCR and DA concentrations. The red curve shows the dependence of NCR on AA concentrations, while the blue curve displays the function of NCR on UA concentrations. The OECTs exhibited a high sensitivity for DA with an LoD as low as 1 nM while maintaining the high selectivity of DA detection against AA and UA in the range of 1 nM to 100 µM.

The surface of the Pt gate electrode was modified by immersing it in the chitosan solution for 30 s and then drying it in air for 24 h at room temperature. The same experimental procedure for evaluating the transfer characteristics of DA, AA, and UA detection was performed using the surface−modified Pt gate electrode. The NCR plots versus the analyte concentrations were obtained from the transfer curves.

Figure 6b displays the relationship between the NCR values and the analyte concentrations after the modification of the Pt electrode when the V_GS_ was 0.4 V and the V_DS_ was −0.3 V. The black curve shows the correlation between NCR and DA concentrations. The red curve displays the relation of NCR on AA concentrations, while the blue curve represents the NCR for UA concentrations. Compared to the results (black curve) in Figure 6a, the OECTs persist in exhibiting an LoD for DA detection as low as 1 nM with the modified Pt electrode. Moreover, the selectivity of DA detection over AA and UA at high concentrations ranging from 10 µM to 100 µM was dramatically enhanced after modifying the Pt electrode with chitosan. In PBS solution (pH = 7.4), chitosan has an isoelectric point (pKa) of 6.4 and holds anionic states, while AA (pKa = 4.2) and UA (pKa = 5.4) are negatively charged. The electrostatic repulsion between AA/UA and the chitosan layer prevents the diffusion of AA/UA through the chitosan layer to the Pt electrode [42]. Therefore, these electrostatic effects in PBS solution can be utilized to improve the selectivity for the detection of DA against AA and UA.

### 3.6. Real-Time Measurement of DA Concentrations with the OECT Microarrays

**Figure 7:**
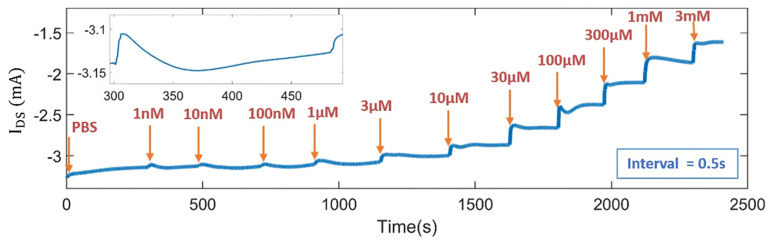
Real-time detection of DA concentrations ranging from 1 nM to 3 mM recorded with time interval of 0.5 s. The inset displays the change in drain–source current when 1 nM of DA was added to the chips. V_DS_ = −0.3 V and V_GS_ = 0.4 V.

Finally, the DA concentrations were monitored in real−time by measuring the drain current while DA solutions of varying concentrations from 1 nM to 3 mM were pipetted onto the chips, respectively. A constant drain–source voltage V_DS_ of −0.3 V and a gate–source voltage V_GS_ of 0.4 V were chosen as the working point. The drain current value was acquired at an interval of 0.5 s. Firstly, the drain current was measured using 50 µL of PBS solution. Subsequently, the drain current was continuously measured in each 50 µL DA solution with concentrations ranging from low to high (1 nM to 3 mM). Figure 7 shows the change in the drain current of OECTs for the real−time monitoring of DA. The inset presents the change in I_DS_ when 1 nM of DA solution was added to the OECTs. As expected from the working principle of PEDOT:PSS OECTs for DA detection, a decrease in the drain–source current I_DS_ was observed with increasing DA concentration. DA solutions with higher concentrations contain more DA molecules, allowing more electro−oxidation of dopamine at the Pt electrode and more Faradic current generation. As a result, a higher effective gate voltage is generated, leading to a larger decrease in I_DS_. Additionally, a significant change in I_DS_ was detected at a DA concentration of 1 nM. These results indicated that OECTs with the Pt electrode as a pseudo−reference electrode can be used as a highly sensitive sensor for DA detection as low as 1 nM.

## 4. Conclusions and Outlook

We have developed a highly sensitive method for the detection of dopamine (DA) using PEDOT:PSS−based organic electrochemical transistor (OECT) microarrays with a Pt electrode as the reference gate electrode. The microfabricated PEDOT:PSS OECT arrays consist of 16 interdigitated electrode (IDE) arrays, with controlled PEDOT:PSS film thickness of 108 nm, 78 nm, and 45 nm achieved by diluting the PEDOT:PSS solution with DI water. We found that reducing the PEDOT:PSS thickness increases the sensitivity for detecting DA concentrations within the range of 1 µM to 30 µM, with OECTs using 78 nm of PEDOT:PSS achieving a low limit of detection (LoD) of 1 nM. These OECTs also showed a sensitivity value in the DA concentration range of 1 nM to 100 µM and high selectivity against ascorbic acid (AA) and uric acid (UA) in a concentration range of 1 nM to 100 μM. By modifying the Pt electrode with chitosan, we were able to significantly enhance the selectivity of OECTs for detecting DA over AA and UA at concentrations ranging from 10 µM to 100 µM. Real−time measurements conducted over a range of DA concentrations from 1 nM to 3 mM showed that the OECTs enable the detection of DA concentrations as low as 1 nM, as evidenced by the change in drain–source current.

The developed platform has the potential for integration within wearable and flexible OECTs for DA sensing applications. However, there are a few important considerations. Firstly, the glass substrate should be replaced with materials that are highly flexible and durable to endure bending and stretching in wearable and flexible applications. Additionally, the sensitivity and selectivity of DA detection based on PEDOT:PSS OECTs in complex physiological and pathological environments should be assessed to ensure their reliability and performance in real−world situations, such as temperature variations, changes in pH levels, and hormonal fluctuations. These microscale PEDOT:PSS OECTs also demonstrate significant potential for various other applications, including in vitro monitoring of cell cultures, neurological research, diagnostic tools, and real-time monitoring of diseases in living organisms.

## Figures and Tables

**Table 1:** PEDOT:PSS-based OECTs for dopamine detection described in the literature.

Gate	Limit of Detection	Sensitivity (Linear Range)	Selectivity	Reference
Pt	5 nM	174 mV/decade (50 nM–3 µM)	-	[44]
Nafion (1.0%)–graphene/Pt	5 nM	281 mV/decade (5 nM–1 µM)	5 nM to 1 µM	[45]
PEDOT:PSS	6 µM	9.1 S M^−1^ (5–100 µM)	0.15 mM	[46]
Nafion/rGO/CSF	1 nM	60 mV/decade (30 nM–10 μM)	1 nM–30 μM	[47]
Pt	30 nM	(30–100 µM)	-	[48]
CNE needle-type	1 pM	(1–160 pM) (2–700 nΜ)	-	[43]
Aptamer-modified Au	0.5 fM	(5 fM^−1^ nM)	10 μM	[49]
o-MIP/Pt	34 nM	-	~0.4 and ~10 μM	[50]
FSP	5 nM	0.899 S M^−1^ (1–6 µM)	50 μM	[51]
Pt	1 nM	0.053/log (M) (1 nM–100 nM) 0.332/log (M) (1 μM–100 μM)	1 nM–100 μM	This work
Chitosan/Pt	1 nM	0.037/log (M) (1 nM–100 nM) 0.343/log (M) (1 μM–100 μM)	1 nM–100 μM	This work

## Data Availability

All relevant data generated or analyzed during this study are included in this published article.
